# Patterns of beta-blocker intensification in ambulatory heart failure patients and short-term association with hospitalization

**DOI:** 10.1186/1471-2261-12-43

**Published:** 2012-06-18

**Authors:** Larry A Allen, David J Magid, Chan Zeng, Pamela N Peterson, Christina L Clarke, Susan Shetterly, David W Brand, Frederick A Masoudi

**Affiliations:** 1 Division of Cardiology, University of Colorado Anschutz Medical Campus, Aurora, CO, USA; 2 Institute for Health Research, Kaiser Permanente Colorado, Denver, CO, USA; 3 Division of Cardiology, Denver Health Medical Center, Denver, CO, USA; 4 Division of Cardiology, University of Colorado Anschutz Medical Campus, Aurora, CO, USA

**Keywords:** Heart failure, Pharmacology, Beta-blocker (β-blocker), Safety, Outcomes

## Abstract

**Background:**

In response to the short-term negative inotropic and chronotropic effects of β-blockers, heart failure (HF) guidelines recommend initiating β-blockers at low dose with gradual uptitration as tolerated to doses used in clinical trials. However, patterns and safety of β-blocker intensification in routine practice are poorly described.

**Methods:**

We described β-blocker intensification among Kaiser Colorado enrollees with a primary discharge diagnosis of HF between 2001–2009. We then assessed β-blocker intensification in the 30 days prior to first hospital readmission for cases compared to the same time period following index hospitalization for non-rehospitalized matched controls. In separate analysis of the subgroup initiated on β-blocker after index hospital discharge, we compared adjusted rates of 30-day hospitalization following initiation of high *versus* low dose β-blocker.

**Results:**

Among 3,227 patients, median age was 76 years and 37% had ejection fraction ≤40% (LVSD). During a median follow up of 669 days, 14% were never on β-blocker, 21% were initiated on β-blocker, 43% were discharged on β-blocker but never uptitrated, and 22% had discharge β-blocker uptitrated; 63% were readmitted and 49% died. β-blocker intensification occurred in the 30 days preceding readmission for 39 of 1,674 (2.3%) readmitted cases compared to 27 (1.6%) of matched controls (adjusted OR 1.36, 95% CI 0.81-2.27). Among patients initiated on therapy, readmission over the subsequent 30 days occurred in 6 of 155 (3.9%) prescribed high dose and 9 of 513 (1.8%) prescribed low dose β-blocker (adjusted OR 3.10, 95% CI 1.02-9.40). For the subgroup with LVSD, findings were not significantly different.

**Conclusion:**

While β-blockers were intensified in nearly half of patients following hospital discharge and high starting dose was associated with increased readmission risk, the prevailing finding was that readmission events were rarely preceded by β-blocker intensification. These data suggest that β-blocker intensification is not a major precipitant of hospitalization, provided recommended dosing is followed.

## Background

β-blocker therapy has emerged as a valuable treatment to reduce mortality and long-term hospitalization in selected patients with heart failure (HF) and left ventricular systolic dysfunction (LVSD) [1-3]. Patients with HF and normal left ventricular ejection fraction (LVEF) may also benefit from β-blockers for heart rate and blood pressure control. As a result, β-blockers are used to treat most patients with HF [4]. However, initiation and uptitration of β-blocker agents produces acute negative inotropic and chronotropic effects, which may lower cardiac output over the short-term [5]. As a result, there are concerns that despite the long-term benefits, β-blockers, initiation or intensification may result in short-term adverse events.

Recognizing these potentially deleterious acute effects of β-blocker use, randomized controlled trials involving β-blockers for the treatment of HF with LVSD have used algorithms where study drug was initiated at a low dose and increased slowly in a supervised manner [6]. Consequently, guidelines recommend, “that β-blockade be initiated at low doses and uptitrated gradually, typically at 2-week intervals in patients with reduced LVEF, and 3–10 day intervals in patients with reduced LVEF following newly diagnosed myocardial infarction.” [2] Existing data suggests that initiation may occur in the ambulatory setting or in the hospital setting [7]. In the controlled environment of a randomized trial, the magnitude of benefit in the first few months of therapy appears to be similar to that seen over the long term [8].

The patterns of initiation and uptitration of β-blockers outside of the carefully monitored confines of randomized controlled trials are poorly characterized. Existing observational data are frequently confined to the inpatient setting or cross-sectional assessments in the outpatient setting. Therefore, concerns remain about the risks of β-blocker use in HF patients with greater comorbidity, particularly without closely-supervised intensification. The limited data that are available show that doses of β-blockers used in clinical practice are on average significantly below doses achieved in randomized controlled trials, which may reflect residual concern about the safety of intensification [9].

To address these gaps in knowledge about the patterns and safety of β-blocker intensification in the real world setting, we evaluated a large cohort of patients with HF cared for in community practice. The goal was to characterize how β-blockers are initiated and uptitrated in patients with HF during routine care and to investigate the association between β-blocker intensification and short-term.

## Methods

### Study setting

All patients in the study cohort were enrolled in Kaiser Permanente Colorado (KPCO), an integrated, nonprofit, managed care organization that provides medical services (inpatient, outpatient, pharmacy) to more than 500,000 persons in the Denver-Boulder metropolitan area through 20 medical offices and several contract hospitals. The study cohort included patients with a primary hospital discharge diagnosis for HF (*i.e.* index HF hospitalization) between January 1, 2001 and July 31, 2009. HF hospitalizations were identified using the principal discharge diagnosis, defined by International Statistical Classification of Diseases and Related Health Problems, 9th edition (ICD-9) codes of 428.xx, 398.91, 402.01, 402.11, 402.91, 404.01, 404.03, 404.11, 404.13, 404.91, 404.93 or a Diagnosis Related Group of 127 (prior to October 2007) and 291, 292, or 293 (October 2007 and beyond), as previously described [10]. Prior literature suggests that a principal discharge diagnosis of HF has a high positive predictive value compared with chart review [11,12]. Following the index HF hospitalization, patients were followed for subsequent events. The study was approved by the Kaiser Permanente Institute for Health Research Institutional Review Board.

Among 4,312 patients identified as having an index hospitalization with a principal discharge diagnosis of HF, we excluded those who did not have a pharmacy benefit through KPCO (n = 10) and those did not survive to hospital discharge (n = 340). In order to allow for a fixed short-term window of exposure time, we excluded those patients who did not have at least 30 days of follow up from the time of index hospital discharge (n = 54) or had readmission in the 30 days after index hospital discharge (n = 681). The resulting cohort included 3,227 patients.

### Exposure to β-blockers

Use of β-blockers was defined as receipt of any of the following oral agents: acebutolol, atenolol, bisoprolol, carvedilol, labetalol, metoprolol succinate, metoprolol tartrate, nadolol, pindolol, propranolol, sotalol, and timolol. These agents represented the available oral β-blockers within the participating health plan and were confirmed based on a search of pharmacy databases for all generic and brand name formulations, including both individual and combination therapies, supplemented by National Drug Codes and American Hospital Formulary Service codes. Of note, there were no formulary restrictions for use of these β-blockers at KPCO during the study period. We used automated pharmacy data on filled outpatient prescriptions to determine the dose and timing, and estimate the duration of receipt of β-blockers based on the days supplied per prescription and refill patterns using a previously established approach [11-13]. The KPCO pharmacy benefit offered prescriptions for a nominal co-payment (typically $5 for a month supply) and prescriptions were conveniently filled at the site of clinical encounters or mailed directly to patients, thus providing a strong incentive to fill prescriptions within the KPCO system. Simultaneous use of multiple β-blockers in the setting of HF is generally not clinically indicated. Therefore, if a patient filled a prescription for a different dose of β-blocker or an entirely different β-blocker drug, the patient was considered to have discontinued the previous β-blocker prescription as of the fill date for the new prescription.

### Data collection

Baseline patient demographics, comorbidities, vital signs and laboratory data were derived from automated KPCO databases. Comorbidities were defined using ICD-9 codes within the automated databases. Data on LVEF was ascertained through manual abstraction of the medical record. The value obtained closest to the index hospitalization was used. LVSD was defined as quantitative LVEF ≤ 40% or qualitative LVEF “moderately” or “severely” reduced. Other HF medications including angiotensin-converting enzyme-inhibitors, angiotensin receptor blockers, and aldosterone antagonists were ascertained using automated pharmacy records using methods similar to those employed for β-blocker exposure. Mortality was ascertained from KPCO databases and validated by comparison with death certificates registered with the State of Colorado. Follow-up and vital status information was available through July 2009. All-cause readmissions were identified from KPCO claims databases.

### Study design

Because β-blocker intensification can occur multiple times for a given subject and at variable times in a subject’s clinical course, a matched case–control analysis [14] was used to assess the association between intensification of β-blocker dose and readmission in the subsequent 30 days. The window of 30-days was chosen based on the known short-term negative chronotropic and inotropic effects of β-blockers in combination with longer-term outcomes data that show neutral 60-day effects and long-term benefit [2,7]. Cases were defined by the first occurrence of hospitalization during follow up (*i.e.* a readmission event). For each case, a control patient was randomly selected among those who had not been admitted to the hospital up to the same amount of time following the index hospital discharge. To increase the number of matched case–control post-discharge time periods, controls could subsequently become cases if they were rehospitalized at a time point further from the index hospitalization than the period for which they acted as a control, provided that an appropriate matched control could be identified. Conversely, cases were not eligible to serve as a control for any time period occurring after their readmission event. In addition to matching the time period from index hospital discharge, control patients were matched with case patients on the basis of the following criteria: age (within 5 years), sex, year of index hospitalization, and presence or absence of LVSD. Cases for which a control could not be identified were excluded from analysis. The exposure was defined as β-blocker initiation or increased β-blocker dose by prescription fill in the 30 days prior to readmission for cases or the 30 days prior to the matched time point for controls.

In contrast to the process of serial intensification of β-blocker doses, initiation of β-blockers is a one-time event. Therefore, we employed a separate cohort design among the subgroup of patients not on a β-blocker during the hospital admission who were subsequently initiated on one. Using this approach we assessed the rate of readmission in the 30 days following β-blocker initiation, comparing high- and low-dose β-blocker. High starting dose was defined above the median initiation dose, and low dose as at or below the median. We then evaluated the association of antecedent high-dose *versus* low-dose β-blocker initiation on rates of readmission in the subsequent 30 days.

In addition to matched variables, the following additional covariates were candidates for adjusted analyses based on clinical significance: diabetes, history of myocardial infarction, history of atrial fibrillation, history of chronic obstructive pulmonary disease, history of chronic liver disease, and systolic blood pressure, heart rate and creatinine clearance preceding readmission for cases or control time period for controls.

### Statistical analysis

Baseline characteristics and medication use were described at the time of index hospitalization using proportions for categorical variables and means with standard deviations (SD) for normally distributed continuous variables and medians and interquartile ranges (IQR) or total range for non-normal data.

We used multivariable logistic regression to evaluate the association of 1) hospital readmission with antecedent β-blocker intensification in the prior 30-days and 2) high-dose *versus* low-dose β-blocker initiation with readmission in the subsequent 30 days. Matching variables were required for inclusion in the case–control analysis. Missing data were <1% for adjustment variables: missing heart rate n = 9, blood pressure n = 9, serum creatinine n = 25. Missing covariates were imputed to the median.

SAS version 9.1 was used for all analyses (SAS Institute Inc., Cary, NC). Statistical significance was determined at the 2-sided alpha = 0.05.

## Results

### Cohort characteristics and overall events

The cohort consisted of 3,227 patients, of whom 37.0% (n = 1,108) had LVSD. The median age of the cohort was 76 years, and 53.2% (n = 1,715) were women (Table 1). The median length of follow-up was 669 days. During follow-up, 62.7% (n = 2,024) patients experienced a hospital readmission for any cause. The median time from index hospital discharge to first readmission was 231 days (range 31–2,814). Almost half (48.7%, or 1,572 patients) died in follow-up.

**Table 1:** Baseline characteristics of the cohort, divided by cases and controls, for the primary case–control analysis

**Characteristic**	**OVERALL**	**CASE Rehospitalized**	**CONTROL Not rehospitalized during same time interval from index hospital discharge**
	**N = 3227**	**N = 1674**	**N = 1674**
**β-Blocker Intensification in Prior 30-Days**		39 (2.3%)	27 (1.6%)
**Matching variables**			
Age, years, median (IQR)	76.1 (67.2-83.5)	75.8 (68.6-82.9)	75.8 (68.5-82.9)
Female Gender	1715 (53.2%)	895 (53.5%)	895 (53.5%)
Year of Index Hospitalization			
2001	409 (12.7%)	229 (13.7%)	229 (13.7%)
2002	393 (12.2%)	220 (13.1%)	220 (13.1%)
2003	362 (11.2%)	222 (13.3%)	222 (13.3%)
2004	401 (12.4%)	247 (14.8%)	247 (14.8%)
2005	438 (13.6%)	261 (15.6%)	261 (15.6%)
2006	354 (11.0%)	186 (11.1%)	186 (11.1%)
2007	358 (11.1%)	176 (10.5%)	176 (10.5%)
2008	344 (10.7%)	123 (7.3%)	123 (7.3%)
2009	168 (5.2%)	10 (0.6%)	10 (0.6%)
LVSD at index hosp	1108 (37.0%)	574 (34.3%)	574 (34.3%)
**Covariates**			
History of Myocardial Infarction	427 (13.2%)	245 (14.6%)	182 (10.9%)
History of Atrial Fibrillation	1422 (44.1%)	759 (45.3%)	748 (44.7%)
History of Diabetes	1457 (45.1%)	836 (49.9%)	732 (43.7%)
History of COPD	1230 (38.1%)	707 (42.2%)	614 (36.7%)
History of Chronic Liver Disease	67 (2.1%)	33 (2.0%)	25 (1.5%)
Systolic BP preceding readmission or matched time period, mmHg, median (IQR)	122 (110–139)	122 (110–138)	124 (110–140)
Diastolic BP preceding readmission or matched time period, mmHg, median (IQR)	68 (60–78)	68 (60–78)	70 (60–78)
Heart rate preceding readmission or matched control time period, bpm, median (IQR)	54 (42–68)	53 (42–67)	55 (44–66)
CrCl preceding readmission or matched control time period, mL/min/1.73 m2, median (IQR)	50.2 (34.9-65.6)	49.8 (34.3-65.1)	55.2 (41.0-71.2)
**β-Blocker Use (at anytime in follow up)**			
Atenolol	551 (17.1%)	307 (18.3%)	334 (20.0%)
Carvedilol	222 (6.9%)	113 (6.8%)	104 (6.2%)
Metoprolol Succinate	526 (16.3%)	266 (15.9%)	261 (15.6%)
Metoprolol Tartrate	1079 (33.4%)	634 (37.9%)	606 (36.2%)
Other β-Blocker	57 (1.8%)	31 (1.9%)	38 (2.3%)
No β-Blocker	792 (24.54%)	322 (19.2%)	331 (19.8%)
Switch β-Blocker Type During Follow Up	538 (2.1%)	343 (25.4%)	311 (23.2%)

*869 controls subsequently became cases due to hospital readmission at a time further from the index hospitalization, and thus are represented in both columns; 748 patients in the “all cohort” did not qualify as a case or a control.

SD = standard deviation; LVSD = left ventricular systolic dysfunction (LVEF < =40% or moderate/severe LV systolic dysfunction); LVEF = left ventricular ejection fraction; COPD = chronic obstructive pulmonary disease; BP = blood pressure; mmHg = millimeters mercury; CrCl = creatinine clearance; mL = milliliter; min = minute; m = meter.

All cells have complete data for the entire cohort, except for blood pressure and heart rate (n = 3,218), and serum creatinine (n = 3,202).

### Characterization of β-blocker use and intensification

During follow up, 14.2% (n = 458) were never on a β-blocker, 21.0% (n = 676) were not on a β-blocker during hospitalization but subsequently started one at discharge or during follow up, 43.0% (n = 1,388) were discharged on a β-blocker but never had the dose increased [of which 1,054 filled this stable β-blocker prescription dose throughout follow up and 334 stopped β-blocker during follow up], and 21.9% (n = 705) had their discharge β-blocker dose increased during follow up. β-blocker type was divided primarily among atenolol, carvedilol, metoprolol succinate, and metoprolol tartrate (Table 1). There was a change from one β-blocker to another during follow up in 22.1% (n = 538) of patients.

Among patients initiated on β-blocker following index hospitalization, the median time from discharge to initiation was 12 days (IQR 1–100) overall, and 7 days and 41 days by LVSD and non-LVSD subgroups, respectively. Among patients discharged on β-blocker whose dose was increased in follow-up, the median time from discharge to first dose escalation was 184 days (IQR 67–532) overall, and 113 days and 266 days by LVSD and non-LVSD subgroups, respectively.

The β-blocker dose achieved at any point in follow up for patients ever treated was a median of 50 mg per 24 hours for atenolol (IQR 25–50 mg), 9.375 mg per 24 hours for carvedilol (IQR 6.25-25 mg), 50 mg per 24 hours for metoprolol succinate (IQR 25–50 mg), and 50 mg per 24 hours for metoprolol tartrate (IQR 25–50 mg).

Among the subgroup with LVSD rates of β-blocker use and uptitration were modestly higher: 9.2% (n = 102) were never on a β-blocker, 32.8% (n = 363) were not on a β-blocker during the index hospitalization but subsequently started one, 31.6% (n = 350) were discharged on a β-blocker but never had the dose increased, and 26.4% (n = 293) had their discharge β-blocker dose increased during follow up (p < 0.001 for comparison to patients without LVSD).

### Relationship of β-blocker intensification to readmission in case–control analysis

Among 2,024 all-cause first readmissions, 1,674 readmitted cases were matched with 1,674 controls. β-blocker intensification was not significantly more common in the 30 days before readmission for cases (2.33%, n = 39) in comparison to a similar time interval for matched controls (1.61%, n = 27; unadjusted odd ratio [OR] 1.44, 95% confidence interval [CI] 0.88-2.36, p = 0.142). After adjustment for history of myocardial infarction, diabetes, atrial fibrillation, chronic obstructive pulmonary disease, chronic liver disease, systolic blood pressure, heart rate, and estimated creatinine clearance at readmission, the association between intensification and readmission remained insignificant (adjusted OR 1.36, 95% CI 0.81-2.27, p = 0.246).

In similar analysis restricted to patients with LVSD (Table 2), β-blocker initiation or uptitration occurred in 2.44% (n = 14) of cases in the 30 days before readmission *versus* 2.26% (n = 13) of non-hospitalized controls during the same post-discharge time interval (adjusted OR 1.13, 95% CI 0.50-2.54, p = 0.77). Conversely, in patients without LVSD, β-blocker initiation or uptitration occurred in 2.27% (n = 25) of cases in the 30 days before hospitalization *versus* 1.27% (n = 14) non-hospitalized controls (adjusted OR was 1.54, 95% CI 0.78-3.04, p = 0.211).

**Table 2:** Absolute and exposure rates to β-blocker intensification for case and control 30- day time periods, with adjusted odds ratios for exposure

	**Proportion with β-blocker intensification in preceding 30-day window**		**Adjusted OR**	**95% CI**	**P Value**
	**CASE (n = 1674)**	**CONTROL (n = 1674)**			
Total	2.33% (n = 39)	1.61% (n = 27)	1.36	0.81-2.27	0.246
LVSD	2.44% (n = 14)	2.26% (n = 13)	1.13	0.50-2.54	0.770
No LVSD	2.27% (n = 25)	1.27% (n = 14)	1.54	0.78-3.04	.221

LVSD = left ventricular systolic dysfunction (LVEF ≤40% or moderate/severe LV systolic dysfunction).

Cases are rehospitalized; controls are not rehospitalized during same time interval from index hospital discharge.

### Comparison of high- *versus* low-dose β-blocker initiation

The starting β-blocker type and dose for the 676 patients discharged from the index hospitalization without a prescription for β-blocker who subsequently initiated therapy with a β-blocker are shown in Table 3. The 8 patients started on labetalol, propranolol, or sotalol were excluded from further analysis. Hospitalization within the 30 days following outpatient initiation of β-blocker occurred in 15 of the 668 patients, 6 of 155 patients (3.87%) initiated on high-dose β-blocker (above the median) and 9 of 513 patients (1.75%) of patients initiated on low-dose β-blocker (median dose or lower). In a multivariable logistic regression model designed to control for other patient-level factors, patients initiated on high-dose β-blocker were more likely to experience readmission in the subsequent 30 days than were those patients initiated on low-dose β-blocker (adjusted OR 3.10, 95% CI 1.02-9.40, p = 0.046). In a sensitivity analysis switching the median dose from the low to the high dose definition, this association between high starting dose and subsequent readmission was partially attenuated such that finding was no longer significant.

**Table 3:** Type and dose of outpatient initiation of β-blockers, stratified by LVEF

**β-Blocker Initiated**	**LVSD (n = 362)**		**No LVSD (n = 283)**	
	**N**	**Dose, mg/24 hr Median (IQR)**	**N**	**Dose, mg/24 hr Median (IQR)**
Atenolol	12	25 (12.5-25)	56	25 (25–25)
Carvedilol	63	12.5 (6.25-25)	12	9.375 (6.25-18.75)
Metoprolol Succinate	124	25 (12.5-37.5)	42	12.5 (12.5-25)
Metoprolol Tartrate	163	50 (25–50)	173	50 (25–50)
Hospitalization in next 30 days following β-blocker initiation (unadjusted)		6 (0.93%)		9 (1.40%)

LVSD = left ventricular systolic dysfunction (LVEF ≤40% or moderate/severe LV systolic dysfunction). The 6 patients initiated on a β-blocker other than those listed were excluded.

## Discussion

β-blocker intensification (initiation or uptitration) occurred in approximately half of patients following index hospital discharge for HF. Using an adjusted matched case–control analysis to better accommodate the short-time window between exposure to β-blocker intensification and readmission, we found no significant increase in exposure to intensified β-blocker dose in the 30 days preceding hospital readmission; although, the low rates of intensification limited the ability to detect even moderate relative differences in readmission rates. In a separate analysis of the subgroup of patients initiated on β-blocker in the ambulatory setting, we found an association of borderline significance between higher starting doses β-blocker and subsequent short-term readmission events. Although the original rationale for the analysis was to test these associations, an equally important finding that emerged from the results was the low absolute rate of β-blocker intensification preceding readmission events (<3% in the prior 30 days) despite initiation and uptitration occurring in nearly half of patients at some point in follow up. This provides objective data that the short-term negative inotropic and chronotropic effects of β-blockers are unlikely to play a major role in the high readmission rates among patients with a history of HF hospitalization.

These data from contemporary clinical practice complement trials data. Randomized controlled trials have generally shown long-term decreases in hospitalization following the use of β-blockers in patients with HF and LVSD, effects which appear relatively early and continue over time [8]. Additionally, initiation of β-blockers during hospitalization for worsening HF in a controlled trial (IMPACT-HF) was not associated with increased readmission at 60 days [7]. The highly controlled, closely monitored setting of randomized trials among a select patient population may favor the beneficial effects of β-blockers while minimizing the potential adverse events. The ratio of benefit to risk in trials may be further optimized with rigid algorithms guiding β-blocker intensification. The applicability of trials to the general care setting is always a concern, especially if many of these safety measures are not routinely employed. Our data show relatively high starting doses for β-blockers and that higher doses were associated with a statistically significant but small absolute increase in readmission. These data thus support guideline recommendations to start β-blocker at low doses and uptitrate gradually.

Heart failure hospitalization and readmission are receiving increasing attention. However, the precipitants of heart failure hospitalizations remain unknown. While various interventions have been proposed to reduce readmission, specific targets for improvement remain elusive. The findings here are relevant to clinical practice and quality improvement initiatives as the low absolute attributable risk of β-blocker intensification for short-term readmission suggests that interventions to improve rates of β-blocker intensification among the LVSD population appear relatively safe. provided guideline recommended dosing is followed.

The overall utilization of β-blockers among patients with LVSD who would be expected to have a guideline indication for β-blocker therapy was 91%. The observed 9% rate of nonuse included nonadherence, as utilization data were derived from pharmacy fills rather than prescriptions. We were unable to perform a more detailed assessment of contraindications to β-blocker use, which might explain a portion of β-blocker non-use. Despite this high overall utilization rate in patients with LVSD, a more detailed assessment of intensification timing and dose maximization of β-blockers suggests potential areas for improvement. The median time from index hospital discharge to dose uptitration was nearly 4 months in patients with LVSD, far longer than is recommended by clinical practice guidelines. Furthermore, the maximum β-blocker doses achieved in this cohort was about half that seen in randomized trials. The reasons for these patterns are unclear. Clinicians may be reluctant to uptitrate β-blocker dose soon after hospitalization for HF decompensation, particularly in a patient population with relatively high levels of comorbidity. However, such reluctance was not seen in the median time to β-blocker initiation (1 week in patients with LVSD), making such an explanation less likely. An alternative explanation is that clinicians are responding to current quality measures which tend to assess whether patients with an indication for a therapy receive it or not, but at present do not typically capture performance on dose maximization.

### Limitations

Certain issues should be considered in the interpretation of this study. First, the retrospective cohort design precluded an assessment of clinical decisions around β-blocker. However, worsening symptoms should generally prompt clinicians to avoid rapid intensification of β-blockers, such that the direction of bias should be towards greater readmission in patients with less aggressive intensification. Because we found the opposite association, this should alleviate some concern for residual treatment selection bias. Second, KPCO is an integrated health organization, and the findings here may not be representative of the fee-for-service setting. Third, we were not able to characterized New York Heart Association functional classification, although we were able to adjust for LVEF and other clinical correlates of HF severity, including blood pressure, heart rate, kidney function, and ischemic heart disease, which are strongly associated with adverse outcomes in HF and rarely available in administrative datasets. Fourth, intensification of β-blockers was determined using pharmacy dispensing that may not capture β-blocker intensification (*e.g.* doubling the dose) that occurred prior to a change in dispensing. Fortunately, such undocumented practices are discouraged in Kaiser’s integrated electronic health records. Fifth, much of the current attention to HF hospitalization has focused on 30-day readmission rates. Because of the need to create a 30-day window to assess pre-hospitalization exposure to β-blockers and because we found that multiple changes to therapies were occurring immediately following index hospital discharge, we had to exclude patients with readmission in these first 30 days. Sixth, our primary analysis looked at all patients with HF, whereas β-blockers have a HF indication only in HF patients with LVSD. However, the majority of patients with HF and preserved ejection fraction are prescribed β-blockers for a variety of reasons, and questions around short-term safety may apply to patients with a wide range of left ventricular ejection fraction. Finally, the study was underpowered to detect small relative differences, and our study is not adequately powered to look carefully at differences by important HF subgroups (*e.g.* LVSD). However, this low power was due in part to a lower than expected intensification rate in the 30 days prior to readmission, which itself became one of the prevailing findings from the data.

## Conclusions

Although β-blocker intensification occurred in nearly half of HF patients following index hospital discharge, readmission events were rarely preceded by β-blocker intensification in the previous 30 days. Higher starting doses may be associated with a relative increase in short-term readmission rates, but the low absolute attributable risk argues that β-blocker intensification patterns are unlikely to be a major contributor to heart failure hospitalization events. Therefore, concern for precipitating hospitalization should not be a major barrier to initiation and intensification of β-blockers for the vast majority of patients with heart failure, particularly if guideline recommendations for β-blocker initiation and intensification are followed.

## Abbreviations

CI, Confidence interval; HF, Heart failure; ICD-9, International statistical classification of diseases and related health problems, 9th edition; IMPACT-HF, Initiation management predischarge: process for assessment of carvedilol therapy in heart failure trial; IQR, Interquartile range; KPCO, Kaiser Permanente Colorado health system; LVEF, Left ventricular ejection fraction; LVSD, Left ventricular systolic dysfunction; OR, Odds ratio.

## Competing interests

Dr. Allen has served as a consultant for Amgen, Janssen Scientific Affairs, and the Robert Wood Johnson Foundation. Dr. Peterson is supported by American Heart Association Pharmaceutical Roundtable grant 067001 N. Dr. Masoudi reported having contracts with the Oklahoma Foundation for Medical Quality and the American College of Cardiology Foundation and serving on an advisory board for Amgen. No other authors reported disclosures or competing interests.

## Authors’ contributions

Dr. LA contributed to the design of the analysis, interpretation of the data, and drafting of the manuscript. Dr. DM contributed to the conception of the database, modifications to the analysis, and critical revision of the manuscript. Dr. CZ contributed by performing the majority of the analysis and through critical revision of the manuscript. Dr. PP contributed to the development of the database, modifications to the analysis, and critical revision of the manuscript. Mrs. CC contributed to creation and modification of the database, modifications to the analysis, and critical revision of the manuscript. Ms. SS contributed to creation and maintenance of the database, modifications to the analysis, and critical revision of the manuscript. Mr. DB contributed to patient privacy and protection issues, management of the database, modifications to the analysis, and critical revision of the manuscript. Dr. DM contributed to the conception of the database, modifications to the analysis, and critical revision of the manuscript. Dr. FM contributed to the conception of the database, modifications to the analysis, and critical revision of the manuscript. All authors have critically reviewed this version of the manuscript and give final approval of the version to be published.

## Pre-publication history

The pre-publication history for this paper can be accessed here:

http://www.biomedcentral.com/1471-2261/12/43/prepub
